# Rethinking Balanced Resuscitation in Trauma

**DOI:** 10.3390/jcm14062111

**Published:** 2025-03-19

**Authors:** Tanya Anand, Hannah Shin, Asanthi Ratnasekera, MyDuyen Luong Tran, Rebekah Huckeby, Lindsey Butts, Ivy Stejskal, Louis J. Magnotti, Bellal Joseph

**Affiliations:** Department of Surgery, Division of Trauma, Surgical Critical Care, Burns, and Acute Care Surgery, University of Arizona, Tucson, AZ 85721, USAasanthi.ratnasekera@christianacare.org (A.R.);

**Keywords:** balanced resuscitation, trauma, endotheliopathy

## Abstract

Hemorrhagic shock from traumatic injury results in a massive systemic response with activation of the hypothalamic–pituitary–adrenal (HPA) axis, pro-thrombotic and clot-lysis pathways as well as development of an endotheliopathy. With ongoing hemorrhage, these responses become dysregulated and are associated with worsening coagulopathy, microvascular dysfunction, and increased transfusion requirements. Our transfusion practices as well as our understanding of the molecular response to hemorrhage have undergone significant advancement during war. Currently, resuscitation practices address the benefit of the early recognition and management of acute coagulopathy and advocates for balanced resuscitation with either whole blood or a 1:1 ratio of packed red blood cells to fresh frozen plasma (respectively). However, a significant volume of evidence in the last two decades has recognized the importance of the early modulation of traumatic endotheliopathy and the HPA axis via the early administration of plasma, whole blood, and adjunctive treatments such as tranexamic acid (TXA) and calcium. This evidence compels us to rethink our understanding of ‘balanced resuscitation’ and begin creating a more structured practice to address additional competing priorities beyond coagulopathy. The following manuscript reviews the benefits of addressing the additional interrelated physiologic responses to hemorrhage and seeks to expand beyond our understanding of ‘balanced resuscitation’.

## 1. Introduction

Traumatic injury remains one of the leading causes of morbidity and mortality in the United States, necessitating prompt interventions to mitigate its life-threatening consequences [1]. At the heart of the management of traumatic injury is resuscitation of the injured patient in hemorrhagic shock [2,3]. Volume resuscitation has focused on restoring circulating blood volume and ensuring adequate oxygen delivery to tissues. With each war, technological advancements and scientific observations of hemorrhaging patients have allowed practitioners to better understand that the transfusion of such patients must resolve additional challenges beyond simply volume replacement and an improvement of perfusion [4,5,6,7]. An additional concern with ongoing or large volume hemorrhage also includes acute coagulopathy; hence the prioritization of balanced (hemostatic) resuscitation [4,5,8,9]. In the last 25 years, the approach to trauma resuscitation has now further evolved due to a better understanding of tissue and microcirculatory biology. Ongoing hemorrhage is also associated with dysregulation of the hypothalamic–pituitary–adrenal (HPA) axis, endothelial and microcirculatory homeostasis, and inflammation [10,11,12,13]. Thus, the emerging evidence begs the question of the true meaning of “balance” in the resuscitation of hemorrhagic shock.

This paper aimed to (1) critically examine the historical evolution of balanced resuscitation in trauma care, (2) explore the current mindset of balanced resuscitation, and (3) reframe “balanced resuscitation” in the hemorrhaging trauma patient beyond simply considering coagulation, but a more expansive and structured practice of addressing dysregulated inflammation, the endotheliopathy of trauma, and imbalance of the HPA axis. By reflecting on the lessons learned from a century of trauma care and highlighting current research, we aim to present a forward-thinking, comprehensive approach to trauma resuscitation that optimizes survival, reduces complications, and enhances recovery in critically injured patients.

## 2. Evolution of Resuscitation: A By-Product of War and Necessity

Over the last 100 years, the dire physiologic circumstances on the battlefield have served as a laboratory in which resuscitation advancements were honed and refined. Prior to World War I, the discovery of ABO blood types and Rh antibodies allowed for improved compatibility in blood transfusions. World War I saw the introduction of citrate preservation, which allowed for cold storage and paved the way for blood banking as well as the rapid administration of blood products [14,15]. By World War II, physicians had pioneered the concept of blood component therapy by separating whole blood (WB) into its components including dried plasma for the resuscitation of traumatic shock [16]. The post-war era ushered in a shift away from whole blood transfusion toward component therapy and crystalloid-based resuscitation, driven by technological advancements in blood fractionation as well as concerns about infectious disease transmission. This trend was further reinforced by influential studies in the 1960s and 1980s that promoted large-volume crystalloid resuscitation for hemorrhagic shock [17,18]. Simultaneously, during the Vietnam War, there was growing recognition of the complications associated with aggressive crystalloid resuscitation such as “shock lung” syndrome or “Da Nang Lung” [18,19]. These clinical findings would be known as acute respiratory distress syndrome [20]. This experience, coupled with mounting evidence of the detrimental effects of excessive crystalloid use, such as acidosis and dilutional coagulopathy, set the stage for a re-evaluation of resuscitation strategies [6].

## 3. Damage Control and the Role of Balanced Fluid Resuscitation

In the late 1990s and early 2000s, the concept of damage control resuscitation (DCR) emerged, drawing lessons from military conflicts in the Middle East and civilian trauma centers [4,21]. DCR emphasizes early hemorrhage control, limited crystalloid use, and balanced blood product administration, aiming to address the “lethal triad” of lactic acidosis, hypothermia, and coagulopathy associated with severe trauma. A 10-year analysis of Operation Iraqi Freedom noted that one-third of patients requiring transfusion had a coagulopathy and benefited from the early transfusion of plasma and platelets [5]. From the 2000s through the 2010s, large-scale studies, such as the PROPPR trial, provided evidence supporting the use of balanced transfusion ratios in massive transfusion protocols [8,17]. Though mortality was not different in patients who had received 1:1:1 (plasma, platelets, and red blood cells, respectively) compared with 1:1:2, there were significant differences in hemostasis achieved between groups [8]. These findings as well as previous studies influenced the widespread adoption of 1:1:1 resuscitation strategies in trauma centers. The most important aspect of the evolution of balanced resuscitation is the increased understanding of the need to manage coagulopathy early [9]. The Prospective, Observational, Multicenter, Major Trauma Transfusion (PROMMTT) study further reinforced the importance of the early administration of plasma and platelet products during the initial hours of care [22]. Thus, concomitantly with managing hypotension, acidosis, and hypothermia, combating coagulopathy became prominent in the traumatologist’s mind.

## 4. Coagulopathy—A Game Changing Consideration

Coagulopathy is noted in nearly a quarter to one-third of hemorrhaging trauma patients and is associated with greater mortality [9,23,24,25]. The need to understand its drivers became clear in the early to mid 1990s during war in the Middle East [5,26]. Early hemorrhage control and correction of coagulopathy were shown to decrease adverse outcomes as ongoing bleeding increases the risk of coagulopathy and overall mortality [9,22,23,24]. Tissue damage and hypoperfusion are inciting factors for coagulopathy [27]. During hemorrhage, mechanisms responsible for both clot formation and fibrinolysis promotion are also ongoing, leading to a consumption of factors [28,29].

Tissue injury damages the endothelial surface with release of subendothelial type III collagen and tissue factor (TF). These factors bind to von Willebrand factor (vWF), platelets, and activated factor VII. The activated TF and factor VII (FVII) complex promotes plasma coagulation proteases and leads to thrombin and fibrin formation. Hyperfibrinolysis is a direct consequence of tissue injury and hypoperfusion [28]. Endothelial injury may increase fibrinolysis and release tissue plasminogen activator (tPA). TPA expression by the endothelium may be increased in the presence of thrombin, which inhibits plasminogen activator inhibitor-1 (PAI-1) and accelerates fibrinolysis. Fibrin is more susceptible to cleavage by plasmin. Activated protein C (APC) is set in motion by thrombomodulin, which is released from the endothelium and binds to thrombin. APC can also inhibit PAI-1. APC inhibits FVa and FVIIIa, which decreases thrombin generation, leading to an anticoagulant state [30].

Systemic hypoperfusion is a major driver for coagulopathy in trauma (COT) [27,31]. Hypoperfusion leads to tissue ischemia and acidosis, causing alterations in the endothelium and thrombomodulin-activated protein C pathway [31]. APC and the consumption of PAI-1 creates a hyperfibrinolytic environment [32]. Both high thrombomodulin and low protein C levels are associated with increased mortality [31,32]. Acidosis caused by a low-flow state may alter the activity of coagulation factors. Meng et al. demonstrated that a reduction in pH from 7.4 to 7.0 significantly decreased the FVIIa and FXa/FVa complex activity, which activates prothrombin to thrombin [33]. Furthermore, animal studies demonstrated acidosis impaired coagulation by depleting clotting factors, inhibiting thrombin generation, and affecting clot strength and stability [24].

Hypothermia after traumatic injury also impacts platelet sequestration and activity as well as coagulation factor activity [33]. The activity of the TF or FVIIa complex decreases with temperature and was only shown to retain 50% of overall activity at 28 °C [33]. Platelets are susceptible to reduced activity with hypothermia due to a reduced effect of von Willebrand factor [33,34]. Meng et al. suggested that bleeding at mildly reduced temperatures (33–37 °C) resulted primarily from a platelet adhesion defect rather than reduced enzyme activity or platelet activation. At temperatures below 33 °C, however, reduced platelet function and enzyme activity led to coagulopathy [34].

With hemorrhage and hypoperfusion, several other important systems are also affected. In the last 25 years, increased research has shown that the endothelium, the hormonal–pituitary axis, and the inflammatory cascade are key mediators in the systemic response to traumatic injury.

## 5. Additional Balanced Resuscitation Considerations

### 5.1. Endotheliopathy of Trauma

The endothelium is a hub for the multisystem coordination of activity and is covered by a gel-like layer known as the glycocalyx [35]. The glycocalyx is negatively charged and composed of glycoproteins and proteoglycans. This unassuming layer has a multitude of functions such as protecting the underlying endothelium from platelet and cell binding, functioning as a mechanotransducer, and regulating the permeability of the underlying endothelium [36,37]. When traumatic injury occurs, the glycocalyx layer is disrupted and sheds its components, leading to a multitude of local, regional, and systemic downstream effects [36,38,39]. There are multiple proxy indicators for glycocalyx injury and the endotheliopathy of trauma (EoT) including syndecan-1 (sdc-1) and soluble thrombomodulin (sTM) [39,40,41]. The shedding of the glycocalyx and damage to the underlying endothelium results in fluid extravasation, edema, leukocyte and platelet adhesion, hyper and/or hypocoagulability as well as impairment in the microcirculation [42]. Together, this is known as the endotheliopathy of trauma and can lead to multisystem organ failure, depending on the severity of injury [38,39]. The disruption and shedding of the glycocalyx propagates a local, regional, and systemic loss of homeostatic regulation of the microvasculature, increases overall inflammation, and mediates the coagulopathy and worsening hemorrhage after traumatic injury [43,44,45]. Targeting this response early during trauma resuscitation has been shown to modulate the overall resuscitation requirements, decrease morbidity, and overall mortality [42,46,47,48,49].

### 5.2. Inflammatory Response After Traumatic Injury

A systemic inflammatory response is activated after traumatic injury, which is dependent on the severity of tissue injury, glycocalyx disruption, and sympathoadrenal activation [41,50,51]. The major occurrence is a locoregional (mild injury) or systemic (severe injury) inflammatory response to ischemic/reperfusion (I/R) injury that generates a cascade of events [52]. If the injury is severe enough, multiorgan failure (MOF) is the result [52]. As noted earlier, other systems propagate the inflammatory response. Transmembrane proteins in the glycocalyx (syndecans) are shown to regulate leukocyte migration and cytokine function [53]. Systemic inflammatory response after injury activates coagulation proteases, which then activate the complement system. Endothelial activation of the thrombomodulin-protein C pathway and competitive binding of C4b binding protein to protein S may lead to alterations in the anticoagulant pathways [54,55]. Modulation of the inflammation after traumatic injury and hemorrhage has been shown to potentially improve survival [56,57,58].

## 6. Hypothalamic–Pituitary–Adrenal (HPA) Axis in Traumatic Injury

After traumatic injury, there is a massive catecholamine release from the adrenal glands and activation of the HPA axis [11,59,60,61]. The hormonal response or activation occurring after traumatic injury and/or moderate to severe hemorrhage activates multiple areas systemically, particularly the inflammatory cytokine cascade, glycocalyx, and endothelial responses in addition to an increased coagulation and a hyperfibrinolytic state [59,62]. With hemorrhage, the HPA axis undergoes significant changes in the secretion of adrenocorticotropic hormone (ACTH) and arginine vasopressin (AvP), also known as antidiuretic hormone (ADH) [11]. Joseph et al. showed that there is an increase in cortisol levels with a concomitant decrease in the ACTH and ADH levels [11]. Sympathetic hormone release is also a key response. By causing a chemical sympathectomy, Xu et al. showed a significant suppression of proinflammatory cytokines, a decrease in glycocalyx shedding, and underlying endothelial injury [62].

Vasopressin, created in the hypothalamus and secreted by the posterior pituitary, is released during hemorrhagic shock in response to hypotension in order to increase systemic vascular resistance and coronary perfusion pressures while not affecting pulmonary vascular resistance [60,63,64,65,66,67]. In other words, it redirects blood flow centrally from the periphery [66,68]. Exogenous vasopressin use is advocated by many animal studies, numerous case reports, and prospective randomized trials [61,68,69]. Although vasopressin is initially rapidly released in response to shock, its secretion dwindles over time, leading to a catecholamine resistant shock [65,70]. Patients with ongoing hemorrhage may have vasopressin deficiency, especially those receiving greater than 5 units of packed red blood cells [71]. A swine study conducted by Gazmuri et al. demonstrated that vasopressin increased systemic vascular resistance by approximately 50% and mean aortic pressure by 10 to 20 mm Hg, concluding a survival benefit with early and sustained vasopressin infusion during severe hemorrhagic shock [72]. A randomized, double-blinded placebo-controlled clinical trial performed by Sims et al. demonstrated an improvement in total blood volume transfused in the first 48 h in patients who received vasopressin compared with those who received the placebo. However, the authors were not able to demonstrate any differences in vasopressor requirements, complications, or 30-day mortality [69].

A number of case reports, animal studies, and at least two prospective human studies have recommended the utilization of vasopressin in hemorrhagic shock [65,68,70,72]. Conversely, a retrospective study by Sperry et al. cautioned the use of vasopressin and other pressors [73]. However, this study was a secondary analysis of a study not designed to test their hypothesis of early vasopressors versus early crystalloid resuscitation. In addition, the utilization of vasopressin should not be used at the exclusion of blood products. Instead, its utilization is important as an adjunct to blood product resuscitation.

## 7. Targeted and Balanced Resuscitation

**Figure 1:**
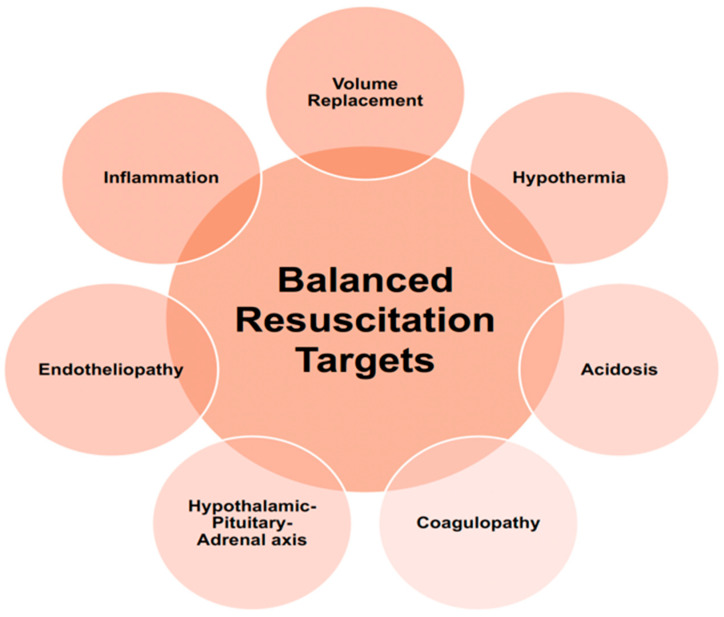
New balanced resuscitation considerations.

The paradigm of “balanced” resuscitation is undergoing an evolution as our understanding of the molecular and microvascular changes after tissue injury and hemorrhage become clearer. As noted, there are concomitant changes that occur with volume loss and tissue injury beyond coagulation abnormalities and acidosis. Current resuscitation practices rely on a few notable measurable endpoints that account for the adequacy of perfusion and assessment of coagulation [71,74,75]. However, there are real-time endpoints (unmeasurable at least for now) that are equally important to modulate during the initial resuscitation [76]. Evidence shows that adapting resuscitation to decrease glycocalyx shedding, inflammation, and hormonal replacement allows for a comprehensive approach to address multisystem derangements more effectively [42,48,49,72,77]. In Figure 1, an expanded paradigm of balanced resuscitation is illustrated. Traditional considerations of what constitutes ‘balance’ must be expanded beyond coagulopathy, hypothermia, acidosis, and hypovolemia to encompass endotheliopathy, inflammation, and changes within the HPA axis.

## 8. Balanced Resuscitation Revamped—Resuscitation Adjuncts and Whole Blood

### 8.1. Tranexamic Acid

There are adjuncts to resuscitation that are associated with a modulation of the EoT [10,42,46,48,60,77,78]. Tranexamic acid (TXA) has been used to limit post-operative blood loss, bleeding related to factor deficiency, and menorrhagia [79]. It maintains clot strength and decreases early breakdown of a formed clot by inhibiting plasminogen binding to plasmin [79]. In the setting of injury and hemorrhage, the early administration of TXA has been shown to decrease the severity of the EoT in both animal and human studies [10,48,77]. Modulation of EoT severity is associated with decreased morbidity and mortality, especially in moderate to severe injury [78,80]. In 2010, a large randomized, placebo controlled trial (CRASH-2 trial) conducted in 40 countries and including over 20,000 patients published the effects of TXA in trauma patients who were bleeding or at-risk of bleeding [81]. The relative risk of death was significantly reduced in patients receiving TXA. Patients did not undergo viscoelastic testing to determine whether they would receive TXA. If bleeding patients were given TXA within 3 h of traumatic injury, then their risk of death was reduced by a third [81]. Additional evidence indicates the safety and benefit of the pre-hospital administration of TXA in patients with suspected or known hemorrhage within three hours of traumatic injury [82,83,84,85]. Several prehospital providers around the world, including those in Nova Scotia, Queensland, Israel, and in the United States, have TXA as part of a standardized protocol in patients who are bleeding or at-risk of bleeding [83]. It is also listed on The World Health Organization’s list as an essential medicine [83].

### 8.2. Calcium

Calcium is another adjunct that plays a vital role in patients with severe trauma and moderate to severe hemorrhage. Nearly 60% of severely injured trauma patients have been shown to present with hypocalcemia [86,87]. It serves as an important mediator of coagulation and the maintenance of glycocalyx thickness in I/R injury [88,89,90]. Ionized or unbound calcium (iCa^2+^) levels decrease during hemorrhage and blood product resuscitation, especially within the first three hours [87,91,92,93]. If persistently low, platelet and clotting factor function, vascular reactivity, and the severity of the resulting endotheliopathy secondary to I/R injury are negatively impacted [91,94]. To minimize the occurrence of hypocalcemia, consideration should be made toward early empiric and/or a protocolized replacement, as even higher doses of calcium are associated with a survival benefit [95,96]. In a study by Wade et al., patients who underwent the protocolized administration and monitoring of calcium levels during resuscitation experienced less hypocalcemia compared with patients who did not undergo regular monitoring [95].

### 8.3. Whole Blood, Components, and Concentrates

#### 8.3.1. Factor Concentrates

In the last decade, there has been an increased interest in the early administration and prioritization of whole blood, factor replacement via plasma and its concentrates, and fibrinogen replacement via cryoprecipitate and its concentrates [78,97,98,99]. The primary interest stems from evidence that the early infusion of plasma and its derivatives (lyophilized plasma, cryoprecipitate, etc.) serve a significant role in modulating the endotheliopathy and coagulopathy that develops with severe traumatic injury and hemorrhage [78,97,98]. Early infusion of these factors and concentrates is also associated with decreased resuscitation requirements as well as resulting morbidity and mortality from traumatic injury [78,100]. Cryoprecipitate is derived from plasma and contains factor VII, factor XIII, vWF, fibrinogen, and other plasma proteins [101]. Early use of cryoprecipitate has been shown to have benefits in traumatic brain injury (TBI) and pediatric patients [102,103]. However, there is conflicting data for the early infusion of cryoprecipitate in injured adults [104,105]. Regarding fibrinogen concentrates, there are a number of metanalyses indicating a potential benefit for its early use [98,106]. Human trials show a measurable increase in fibrinogen levels when delivered early, with conflicting evidence regarding mortality and morbidity benefit as well as some concern regarding the feasibility of delivery during early resuscitation [107,108,109,110].

#### 8.3.2. Fresh Frozen Plasma

Regarding the use of fresh frozen plasma (FFP), freeze-dried (lyophilized), or factor concentrates, there are more consistent data showing a benefit in their early utilization in trauma resuscitation [99,105,111,112,113,114]. These benefits include modulation of the inflammatory response and overall resuscitation. Important considerations that must be studied further are the optimal timing and administration order of factor concentrates, plasma, and plasma concentrates within a massive resuscitation protocol that will yield the greatest benefit for balanced resuscitation [42,97,104,105].

## 9. Whole Blood

Whole blood, a staple of resuscitation in hemorrhagic shock during World Wars I and II, is witnessing a resurgence [115,116]. Though whole blood has been used since World War I, component therapy became more popular between the 1940s and 1980s because of the flexibility in storage and the ability to transfuse separate products based on need [116]. In the last two decades, however, there has been a shift in the prioritization of WB, as early WB administration is associated with improved outcomes and decreased overall volume required for resuscitation [117,118,119,120]. Some of the reasons for this paradigm shift include the simplification of resuscitation logistics, a decrease in infused preservative volumes, and a more efficient delivery of improved ratios of red blood cells, plasma, and platelets [121]. In addition, component therapy has 40% fewer coagulation factors than WB and has more additives such as dextrose, mannitol, and citrate [122]. Some systematic reviews comparing WB transfusions to blood component therapy have shown decreased overall transfusion volume, while others have shown no change in the overall transfusion volume [120,121,123]. In terms of cost, WB is cited to decrease the cost associated with transfusions [124,125]. These cost savings are likely secondary to the decreased processing of WB compared with component therapy, decreased storage cost, and decreased waste [124,125].

## 10. Balanced Resuscitation Protocol

The evidence discussed, thus far, now brings about a dilemma on how to proceed with resuscitation with the additional considerations in a more structured protocol. It is important to create a functional algorithm that guides the thought process behind resuscitation and is malleable if a resource poor site (i.e., a small community trauma center) does not have access to certain products (i.e., whole blood) or has limited capabilities. We discussed additional considerations in traumatic hemorrhage for truly balanced resuscitation as well as the role of various adjuncts and components in targeting these changes. We also focused on the importance of considering the HPA axis and ensuring that baseline vascular tone is prioritized during resuscitation.

**Figure 2:**
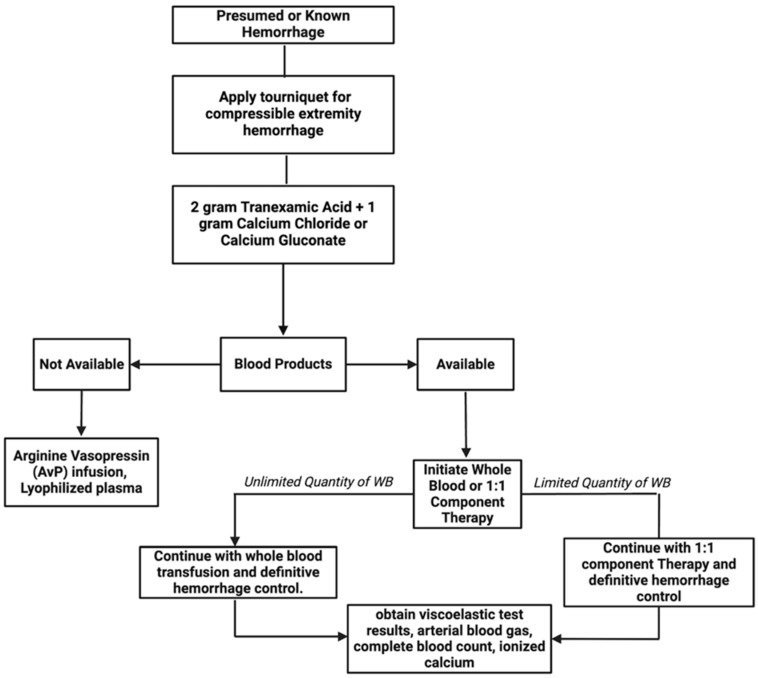
Approach to protocolized resuscitation in hemorrhagic shock.

We propose a basic guideline (Figure 2) for a protocolized approach to balanced resuscitation that may guide the practitioner, even in a resource poor setting. Because timely treatment is vital in a bleeding patient, suspicion of hemorrhage is sufficient to begin utilization of this protocol. With compressible bleeding, the application of a tourniquet for hemorrhage control should be prioritized. The recommended next step is to address hypocalcemia as well as the coagulopathy, inflammation, and endotheliopathy through the early administration of TXA. If blood is available, then transfusion of either WB or a 1:1 PRBC to FFP resuscitation should occur concomitantly with TXA and calcium delivery. If blood products are not immediately available and bleeding is non-compressible, then measures to centrally direct blood flow with an arginine vasopressin infusion and addressing coagulopathy and endotheliopathy with lyophilized plasma may be considered.

Potential barriers to implementation include both cultural and infrastructure barriers [122]. The acquisition of blood products may take time and may not be readily available. This protocol takes the potential delay in the availability of blood products into account and supplements resuscitation with adjuncts that will maintain vascular reactivity and provide early modulation of the inflammatory, endothelial, and coagulation responses to hemorrhage. Other infrastructure barriers include varying pre-hospital expertise and resources [126].

## 11. Special Populations

### 11.1. Pediatric Patients

The principles of hemostatic resuscitation in injured pediatric patients are largely consistent with injured adults [127]. The primary goals of minimizing hypothermia, acidosis, and hypocalcemia remain the same. However, recognizing that an injured child is in the early stage of hemorrhage is often difficult, given their remarkable ability to maintain circulation even with a 40% loss of blood volume [128]. In terms of blood product resuscitation, whole blood in pediatric trauma patients have shown decreased overall transfusion requirements [129,130,131]. The evidence favors 1:1 resuscitation rather than increased early plasma resuscitation [130,131]. In terms of the utilization of adjuncts such as TXA, more evidence is needed. A systematic review that included six single institution and eight multicenter studies showed no statistically significant increase in survival in the civilian setting. Increased survival was noted in children receiving TXA in the combat setting [132]. Other studies, however, have not shown the resounding benefit observed in injured adults [133,134,135]. In fact, one study cited an increased incidence of seizures [133].

### 11.2. Injured Older Adults

Physiologic response to traumatic injury and hemorrhage have been shown to differ in older adults [76,136]. The altered response is likely secondary to age-related changes in physiology and the impact of multiple medications upon compensatory mechanisms [136,137,138]. Certain medications, such as beta blockers, may lead to an atypical presentation of hemorrhagic shock and a delay in recognition [137]. Other medications, such as anticoagulants, may require the practitioner to consider the administration of reversal agents (i.e., prothrombin complex concentrate), FFP, and/or platelets to decrease the severity of bleeding and promote hemostasis [139,140]. In terms of transfusion, WB, concomitantly with component therapy, has been shown to benefit injured older adults and is associated with improved early mortality and decreased morbidity [141,142]. TXA has primarily been studied in older adults with hip fractures and has been shown to decrease blood loss and the overall transfusion requirements [143,144].

## 12. Conclusions

Traumatic injury and hemorrhage induce a complex response that simultaneously activates multiple systems. Research in the last 25 years has shown that endothelial injury, HPA axis changes, and overall inflammation during hemorrhage are additional foci to target during resuscitation. To underestimate the importance of a particular system is to advocate for an “imbalanced resuscitation”, as each system is intricately linked. A more balanced approach to resuscitation involves the inclusion and conscientious incorporation of resuscitation practices that modulate and support these additional systems. Key recommendations are summarized in Table 1.

## 13. Future Perspectives

Additional studies are being conducted with the aim to improve our understanding of the impact of various adjuncts and biomarker changes in addition to blood product resuscitation. **The CAlcium and VAsopressin following Injury Early Resuscitation trial (CAVALIER)** and **bioTROOP** trials are examples of such studies. Other studies, such as the Massive Transfusion in Children-2 (MATIC-2) trial, are studying the impact of WB versus component therapy in pediatric hemorrhage. Further research needs to investigate the endothelial response to trauma, the HPA axis changes, and how they are interrelated in certain subpopulations to better understand how to prescribe adjunctive therapies in older adults and pediatric patients. The aim to create a truly balanced resuscitation algorithm is fast becoming a reality and will further delineate a more specific resuscitation algorithm.

## Figures and Tables

**Table 1:** Key Summary Concepts for Balanced Resuscitation.

Key Recommendations
**Considerations of endotheliopathy, inflammation, and dysregulation of the HPA axis are equally important considerations in the hemorrhaging trauma patient.** **The incidence of hypocalcemia in moderate to severe trauma is over 50%.** **TXA is safe and effective when administered early during suspected or severe hemorrhage in injured adults.**
